# Optimisation tools for meeting nutrient requirements of Indian children and adults at optimal cost

**DOI:** 10.1017/S1368980025100748

**Published:** 2025-07-16

**Authors:** Fathima Ayoob, Jawahar R. Manivannan, Ashikh Ahmed, Afsal K. Murikkanchery, Abhishek Kumar, Santu Ghosh, Patrick Webb, Anura V. Kurpad, Tinku Thomas

**Affiliations:** 1 Division of Epidemiology and Biostatistics, St John’s Research Institute, Bengaluru, India; 2 Department of Biostatistics, St John’s Medical College, Bengaluru, India; 3 Friedman School of Nutrition, Tufts University, Boston, MA, USA; 4 Department of Physiology, St. John’s Medical College, Bengaluru, India

**Keywords:** Optimisation, Nutrition requirements, Optimal cost

## Abstract

**Objective:**

To present a tool and examine the minimum cost of a healthy and diverse diet that meets the daily requirements of essential nutrients for the people of India, using interactive web-based tools.

**Design:**

Linear-programming algorithms were adapted into two web-based tools: a Food Optimisation for Population (FOP) tool and a Diet Optimisation Tool (DOT). The FOP optimises daily food choices at a population level, considering local food consumption patterns. The DOT focuses on household or individual food selection.

**Setting:**

India, with consideration of locally produced and consumed foods.

**Participants:**

The two optimisation tools are demonstrated for the state of Bihar: the FOP tool at the population level, exemplified by diet optimisation for children aged 1–3 years, and DOT at the household level, demonstrated through diet optimisation for a household of four members.

**Results:**

Both tools provide cost-effective, optimised food plans, respecting cultural preferences. Based on food prices from June 2022, the FOP tool generated optimised diets for 1–3-year-old Bihari children priced at INR 26·8 (USD 0·32 converted as of January 2024 rate)/child/day. By applying a milk subsidy, this cost could drop to INR 23·7 (USD 0·28). The DOT was able to formulate a vegetarian diet for a family of four at INR 204 (USD 2·45)/day.

**Conclusions:**

These web-based tools offer diet plans optimised to meet macro- and micronutrient requirements at population and/or individual/household levels, at minimum cost. This tool can be used by policymakers to design food-focused strategies that can meet nutritional needs at local price points, while considering food preferences.

Chronic undernutrition is primarily associated with the nutritional value of food rather than with its quantity^(1)^. Diet diversity has a critical role in meeting nutrient adequacy, which in turn can reduce the risk of conditions like stunting, wasting and underweight in children^(2)^, which carry elevated mortality risks^(3)^. This linkage has been observed in adults as well^(4–6)^.

To achieve appropriate food and nutrition security, it is critical to diversify diets with adequate quantities of fruits, vegetables and animal-source foods, particularly to prevent common micronutrient deficiencies like vitamin A, Fe and Zn^(7)^. The cost of a recommended diverse diet for adult Indians was estimated to be unaffordable for 63–70 % of the rural poor in India^(8)^. One way to influence dietary diversity is through consumer price subsidies on nutritious foods such as fruits and vegetables^(9)^. It is also possible that having an increased disposable income due to food subsidies on staples could lead to a higher consumption of diverse foods, but this may not be necessarily true due to higher price of quality foods such as milk, fruits and vegetables – leading to potential diversification into foods that are high in sugar, oil and fat and not deemed to be supportive of good health^(10)^. The Public Distribution System of food under the National Food Security Act^(11)^ is important in ensuring food security of the Indian population by the provision of subsidised food grains to the poor^(12)^. However, the latter has changed food consumption patterns because the provision of rice at subsidised rates shifted consumption patterns away from traditional staples, like millets^(9)^. It can be expected that inclusion of quality foods such as milk, egg or fruits into the programme can potentially change dietary patterns and improve dietary diversity in the population. Efficient design of such programmes requires detailed cost-sensitive and location-specific knowledge of foods as well as knowledge on optimisation algorithms that can identify food combinations that meet nutrient requirements.

Governments should use data on the most economical methods to meet dietary requirements to offer focused assistance aimed at food preferences. This can ensure that citizens have access to nutritious food in adequate amounts for nutrient adequacy^(13)^. However, the policies on provisions and subsidies are often made without adequate research and calculation on what should be provided and in what quantities to meet the nutrient requirements of the population.

In this paper, we describe the development of two linear programming algorithms that optimise food combinations. Both algorithms have been converted to interactive web-based tools. The first algorithm is for optimising food combinations at a population level based on locally consumed and produced foods, also called the Food Optimisation for Population (FOP) tool (https://www.datatools.sjri.res.in/FOP/index). The second algorithm is for optimising food combinations at a household or individual level, also called the Diet Optimisation Tool (DOT) (https://www.datatools.sjri.res.in/DOT/). To demonstrate the features of the tool and the interpretation of its outputs, we examine the minimum cost of a diverse diet at population and sub-group levels to meet macro- and micronutrient daily requirements.

## Methods

The estimated average requirement (EAR) is the average daily intake of nutrients estimated to meet the median nutrient requirement of a healthy population. The EAR is used to plan nutritionally adequate diets for a group of individuals or a population^(14)^. The EAR was used in this study to characterise daily nutrient requirements and to set targets for optimised daily food intake. The EAR is available for Indians of different age and sex groups^(15)^. The tool optimised eighteem important macro- and micronutrients: energy (kcal), protein (g), fat (g), carbohydrate (g), dietary fibre (g), Ca (mg), Zn (mg), Fe (mg), Mg (mg), iodine (µg), vitamin A (µg), folate (µg), vitamin B_12_ (µg), vitamin B_1_ (mg), vitamin B_2_ (mg), vitamin B_3_ (mg), vitamin B_6_ (mg) and vitamin C (mg). Each nutrient’s EAR is presented in online supplementary material, Supplemental Table 1.

### Development of ‘Food Optimisation for Population tool’ and ‘Diet Optimisation Tool’

The algorithm driving the FOP tool was designed to answer the following question: What food combinations must be consumed to meet multiple macro- and micronutrient requirements of a population residing in any geographical part of India, while considering local foods and minimal cost? This algorithm can be used to analyse how food subsidies impact the cost of various food combinations required to meet multiple nutrient requirements.

The optimisation used a database of 120 raw foods habitually consumed in India, categorised into 12 food groups. It facilitated separate optimisations on four basic diet plans: namely, *Diet1* (vegan), *Diet2* (lacto-vegetarian), *Diet3* (lacto-ovo-vegetarian) and *Diet4* (non-vegetarian). The tool proposed an optimised diet based on the user-defined choice of foods for consumers in any Indian state or district. An added feature was built into assess the affordability of the optimised diets for different family compositions, by specifying the number of family members in each age group: ‘1–9 years (child)’, ‘10–18 years (boys and girls)’, ‘adult men’ and ‘adult women’.

The DOT algorithm was primarily designed to answer a slightly different question, as follows: What is the cost of meeting the macro- and micronutrient requirements of an individual or a household, residing anywhere in India, based on a set of food items across different food groups? The DOT considers 52 food items across 12 food groups, but it targets the requirements of 17 macro- and micronutrients (carbohydrates excluded here) with respect to the age and sex of the individual.

### Sources of data

Food composition data were obtained from the Indian Food Composition database^(16)^, except for sugar and olives (obtained from the United States Department of Agriculture’s food composition database)^(17)^. For the FOP tool, population projections were calculated using Indian birth rates, sex ratios, survival rates and fertility rates^(18,19)^, with the 2011 Census data serving as the baseline for projecting the population for the year 2020. These projections were generated using the Dynamic Demographic Projection Model. State-specific population estimates were derived from population counts at the state and district levels. While based on the widely used cohort component method for estimating India’s population, this approach has been refined to account for the dynamic behaviours of fertility, mortality and migration. The resulting Dynamic Demographic Projection Model is a widely recognised and extensively applied tool for population projections.

The list of food items used for the FOP included all those covered in the National Sample Survey Office Consumer Expenditure Survey 68th Round (2011–2012)^(20)^, which captured household food consumption, and from the Area Production and Yield Statistics for the year 2000–2019^(21)^, which captured food availability. These data were used to ensure that the optimisation algorithm made suggestions of foods based on the region and the foods commonly consumed and are available in these regions.

Market prices for the listed food items were obtained from the Agmarknet website^(22)^ for the year 2022, and prices for each commodity were aggregated by their arithmetic mean at the state and district levels. State-specific wage data for assessing the affordability of optimised diets were obtained from the Reserve Bank of India 2020–21 handbook (RBI, tables 96–99 under ‘Prices and Wages’)^(23)^, which provided state-specific average wages per day. The default food prices used in DOT were market prices sourced from the Agmarknet website^(22)^ for June 2022.

### Linear programming

The nutrient optimisation process for both tools was carried out using linear programming. The primary objective of the linear programming model was to find the optimal values for the variables that minimised the cost of the diet. The cost of the diet, as the objective, was expressed as a linear function (equation 1), where, 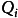 is the quantity of food item 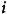 *(g)*, *C*_*i*_ is the cost per gram of raw food item 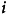 , *N*_*j*_ is the *j* ^th^ nutrient of interest and 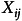 is the *j* ^th^ nutrient content in 1 g of the *i* ^th^ food item.

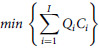

subject to

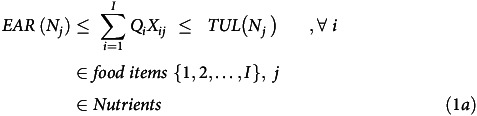




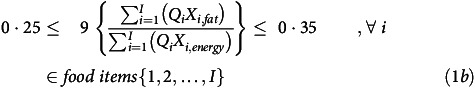




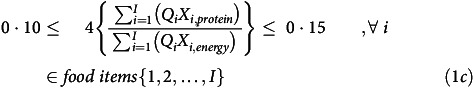




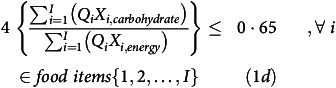




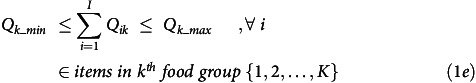




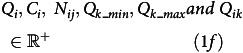





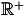
 indicates the set of real numbers

The primary objective function was minimised subject to specific constraints to ensure that age-specific nutritional recommendations for a healthy population were satisfied. For both FOP and DOT, the tolerable upper limit of nutrient intake served as a constraint for the maximum allowable daily nutrient intake (equation 1a). This meant that the optimised diet proposed should have fulfilled the daily EAR for all listed nutrients, while not crossing the tolerable upper limit for any of them.

In addition, the fat-energy ratio in DOT was constrained to be within 25–35 %^(12)^ (equation 1b, where *X*_*i,fat*_ and *X*_*i,energy*_ are the fat and energy value of the *i* ^*th*^ food item), and for FOP, a wider range of fat-energy ratio (35–40 %) was considered for the optimisation as this was a population-level optimisation.

The protein-energy ratio was constrained to be within 10–15 %^(12)^ (equation 1c, where *X*_*i,protein*_ and *X*_*i,energy*_ are the protein and energy value of the *i* ^*th*^ food item) for both DOT and FOP. The carbohydrate-energy ratio was constrained to be within 60–65 %^(15)^(equation 1d, where *X*_*i,carbohydrate*_ and *X*_*i,energy*_ are the carbohydrate and energy value of the *i* ^*th*^ food item).

FOP tool was used to optimise four different meal types, which differed from each other by the inclusion or exclusion of milk and animal-source foods. The constraints for each of the diet types (*Diet1*–*Diet4*) ensured that each diet plan was tailored to meet specific nutritional considerations based on its vegan, vegetarian or non-vegetarian nature and the presence or absence of particular food items.

*Diet1* (vegan) optimised all listed nutrients (see online supplementary material, Supplemental Table 1) to meet their EAR, except vitamin B_12_; for vitamin B_2_ and Ca, the constraint was that only 50 % of EAR could be met. This was because EAR can be met for these nutrients from *Diet1* only if excess quantities of certain foods like green leafy vegetables are consumed. *Diet2* (lacto-vegetarian) and *Diet3* (lacto-ovo-vegetarian) considered meeting the EAR of all nutrients, but specific constraints were set for the need for milk-based foods to meet the EAR of vitamin B_2_ and vitamin B_12_ (equation 1a). *Diet4* (non-vegetarian) considered meeting the EAR of all nutrients; however, if only one animal-source food were considered for this optimisation, then 50 % of EAR was considered as the target for vitamin B_12_. It is possible to meet the EAR of vitamin B_12_ from a single food only if a large quantity of the food is consumed. These specific changes to meeting the requirements of B vitamins were made because specific foods were required to meet the requirements of all B vitamins.

Additional constraints were introduced to ensure dietary diversity by establishing quantity limits within each food group (equation 1e, where *Q*_*k_min*_ and *Q*_*k_max*_ are the minimum and maximum intake quantity^(24)^ for the *k* ^*th*^ food group; these values are age and sex specific, considered with some flexibility for optimisation). An assumption was also made that the recommended intake of iodine was derived solely from iodised salt.

The following additional constraints for DOT were considered to arrive at optimal feasible solutions. The minimum daily quantities of food to be consumed in each of the food groups (green leafy vegetables, milk products, roots and tubers and other vegetables) were defined (equation 1e). The quantity of egg that could be consumed in a day was constrained to 50 g (one egg/day)^(25,26)^ (equation 1e). The minimum daily quantity of the green leafy vegetables was fixed at 20 g and that of other vegetables and tubers set at 30 g (equation 1e). Vitamin B_12_ could be optimised only if animal-source foods were included for optimisation. The quantity of sugar was constrained to 10 % of the total energy intake^(15)^ (equation 1e).

### Optimisation tool

An interactive linear optimisation application (App) for public use is now hosted at https://www.datatools.sjri.res.in/FOP/index and https://www.datatools.sjri.res.in/DOT/. The FOP provides the user with state- and district-specific optimised diet plans with average nutritional breakdown and food intake quantities per day for all age and sex groups: children^(15)^ (1–3, 4–6 and 7–9 years), boys (10–12, 13–15 and 16–18 years), girls (10–12, 13–15 and 16–18 years), men (> 18 years) and women (> 18 years), with costs of an optimised diet, presented per day, week, month or year. The FOP tool also gives the cost for a projected population of the selected location for the year 2020.

The Household Affordability module in FOP allows for an assessment of the notional affordability for households. The calculator has the flexibility to add age and sex specific household members and indicate the number of earning members in the household. The calculator also considers state-specific average wages as per the 2020–2021 RBI Handbook^(23)^. These data are used by the tool to indicate the affordability for each type of diet plan as well as the ratio of food expenditure to wages. This ratio gives the percentage of wage that needs to be spent by an average household for food expenses.

The FOP tool has been demonstrated for the state of Bihar, and the optimisation has been performed for *Diet4*. Two options, including milk at retail price and at 50 % subsidised price, are demonstrated. The state of Bihar was chosen for this demonstration because it has a diverse diet. The age group 1–3 years was chosen because this is a particularly vulnerable group that has additional nutritional requirements due to rapid growth, and they are beneficiaries of a national supplementary nutrition programme. To demonstrate the usefulness of the tool, milk was chosen as a specific food to optimise diets with an eye for costs, because it is an excellent and widely available source of nutrients for this vulnerable age group (1–3 years). It is already included in the supplementary nutrition programmes of several states of India and is also an acceptable food. The DOT provided the average quantity of user-selected raw foods to be consumed by the household, either for a day, week or month, based on the user’s choice, to meet the daily requirement of seventeen different nutrients at minimal cost. In addition, the tool computed the optimised cost of diet for any household composition. Users have the flexibility to optimise for all age groups and all four diets simultaneously, enabling a comparison of optimal costs and intake quantities across diets.

To demonstrate the utility of DOT, an optimisation was performed for a family of four members (male child 1–3 years, female child 4–6 years, adult male > 18 years and adult non-pregnant, non-lactating female 15–45 years) and two members (adult male > 18 years and adult non-pregnant, non-lactating female 15–45 years) for a vegetarian diet without meat and eggs.

Notably, both tools include an interactive feature that allows users to update food prices for any commodity, such as subsidised prices for milk, rice, oil, etc., before conducting the optimisation exercise, enabling scenario-based analyses of the minimum cost of diets.

To evaluate the variability and robustness of the results obtained from the tools, non-converging outcomes were analysed. The analysis revealed the requirement for a minimum number of items to be selected from each food group to meet the requirements of all selected nutrients. This assumption was rigorously tested for variability and robustness across both tools, and appropriate constraints to achieve convergence in optimisation were developed.

Python version 3.8.3 and the ‘PuLP’ package were used to arrive at an optimal diet for both applications. These were developed using the Django Framework (Python) and HTML, CSS and JavaScript for the frontend.

## Results

**Fig. 1.**
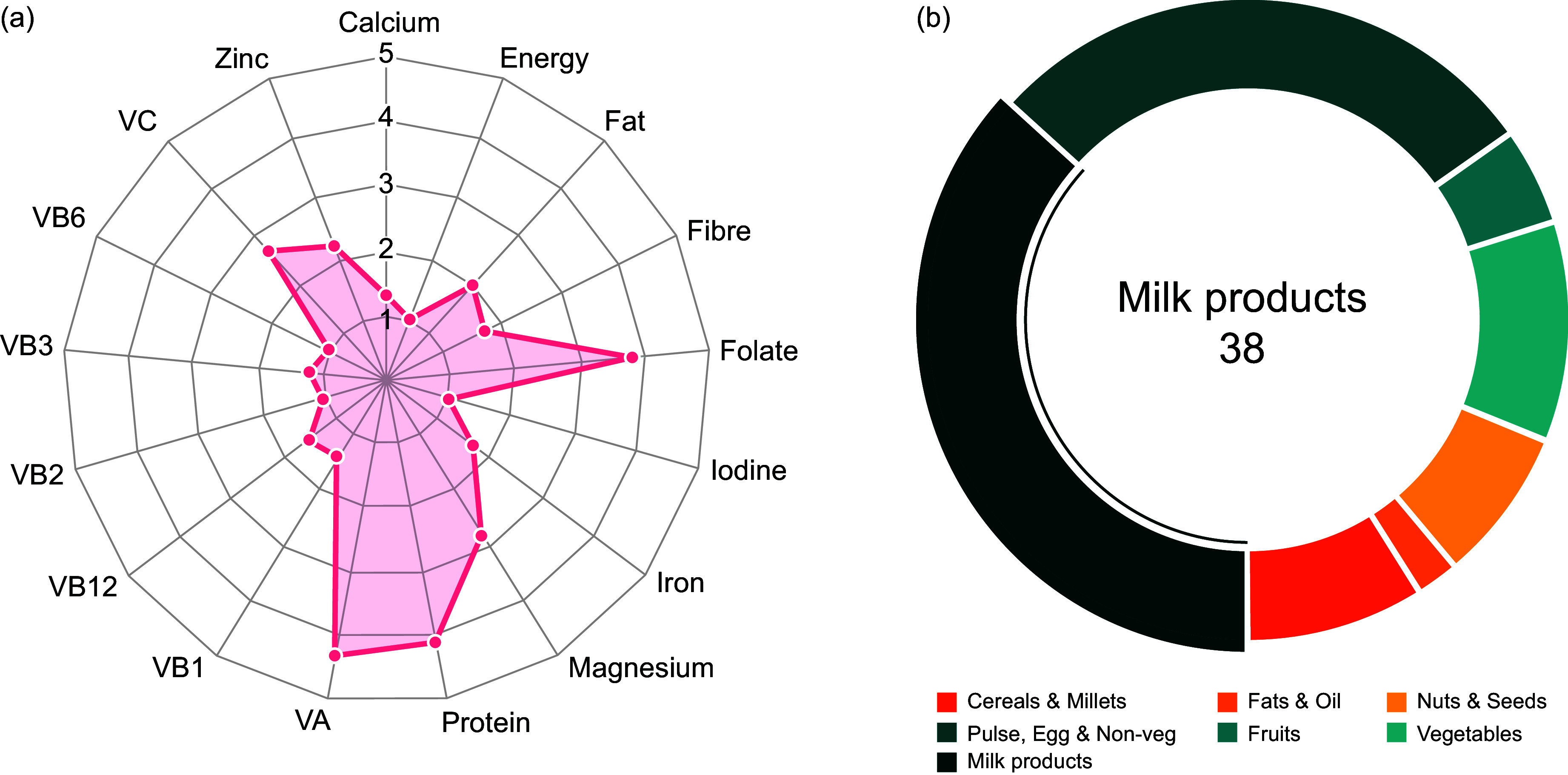
Visual representation of an optimised diet example, with subsidised milk, for children aged 1–3 years in Bihar, using the FOP tool (https://www.datatools.sjri.res.in/FOP/index) . (a) A radar chart displays the proportion of different nutrients in an optimised diet relative to their recommended values. A line at 1 signifies that the optimised nutrient amount matches the recommended intake, indicating a ratio of 1. (b) The doughnut chart segments represent different food groups, showing the percentage each group contributes to the optimised diet. When using the online tool, hovering over a segment with the cursor reveals additional details.

To demonstrate the FOP tool, the state of Bihar was chosen, and a non-vegetarian diet (*Diet4*) was optimised for a projected population of 1- to 3-year-old children living in Bihar state of India. An optimal diet plan that fulfils nutritional requirements while satisfying all constraints was obtained. Figures from the FOP tool provide details of the optimised solution, described as follows. The Radar chart (Fig. 1(a)) illustrates the ratio of each nutrient intake to its recommended value, providing a quick visual representation of the adequacy of the diet plan. Additionally, Fig. 1(b) presents the distribution of each food group in a daily diet plan, that is, the intake percentage of each food group. The cost of one optimised solution from a selection of foods for children (age 1–3 years) of Bihar, with milk at retail price (cost of milk is INR 50/litre) was INR 26·8/day/child (Prices in June 2022 used, USD rate as of January 2024; USD 0·32); when subsidised cost of milk was considered (subsidised cost of milk is INR 25/litre), the optimised cost of *Diet4* reduced to INR 23·7/day (USD 0·28/day). The cost of diet decreased by INR 3/child/day (Table 1) by subsidising milk, while dietary Ca increased by 98 mg/child/day (Table 2). Specifically, Table 1 presents the cost of the diet, while Table 2 presents the nutrient composition of the optimised diet. Table 3 presents the food group composition of the optimised diet.

**Table 1.** Cost of optimised diet for children aged 1–3 years in Bihar^*^

Population	Cost of diet with market price of milk		Cost of diet with subsidised milk^†^	
	Cost for one child *n* 1	Cost for all children^‡^ *n* 7·426 (million)	Cost for one child *n* 1	Cost for all children^‡^ *n* 7·426 (million)
Per day	INR 26·8	INR 198 940 611·1	INR 23·7	INR 175 623 197·2
Per week	INR 187·5	INR 1 392 584 277·8	INR 165·5	INR 1 229 362 380·4
Per month	INR 803·7	INR 5 968 218 333·6	INR 709·5	INR 5 268 695 916·0
Per year	INR 9778·4	INR 72 613 323 058·8	INR 8632·3	INR 64 102 466 978·0

* Demonstration example generated using FOP tool (https://www.datatools.sjri.res.in/FOP/index).

† Market price of milk subsidised from INR 50/litre to INR 25/litre.

‡ All children in the state of Bihar, India.

**Table 2.** Nutrient composition of optimised diet for children aged 1–3 years in Bihar^*^

Nutrient	Nutrient composition (market price of milk)	Nutrient composition (with subsidised milk)^†^
Ca (mg)	411	509
Carbohydrate (g)	99	99
Dietary fibre (g)	27	25
Energy (kcal)	1010	1010
Fat (g)	51	50
Folate (µg)	324	346
Iodine (µg)	65	65
Fe (mg)	10	10
Mg (mg)	325	313
Vitamin B_3_ (mg)	9	7
Protein (g)	38	38
Vitamin B_6_ (mg)	0·8	0·8
Vitamin B_2_ (mg)	0·8	0·8
Vitamin B_1_ (mg)	0·9	0·8
Vitamin A (µg)	616	774
Vitamin B_12_ (µg)	1	1
Vitamin C (mg)	47	58
Zn (mg)	6	6

* Demonstration example generated using FOP tool (https://www.datatools.sjri.res.in/FOP/index ).

† Market price of milk subsidised from INR 50/litre to INR 25/litre.

**Table 3.** Food group composition of optimised diet for children aged 1–3 years in Bihar^*^

Food name	Food group	Quantity per day (g) (market price of milk)	Quantity per day (g) (with subsidised milk)^†^
Wheat flour atta	Cereals and millets	60	60
Bengal gram whole	Pulse	20	20
Spinach (Palak)	Green leafy vegetables	72	100
Bathua leaves	Green leafy vegetables	28	–
Oil	Oil	10	10
Salt	Salt	2	2
Sugar	Sugar	26	24
Brinjal	Vegetables	100	100
Radish	Roots and tubers	30	30
Mango	Fruits	30	30
Groundnut	Nuts	42	26
Coconut green	Nuts	23	25
Green chillies	Spices	5	5
Cow milk	Milk products	139	260
Egg	Egg	45	45
Chicken	Non-veg	10	10

* Demonstration example generated using FOP tool (https://www.datatools.sjri.res.in/FOP/index ).

† Market price of milk subsidised from INR 50/litre to INR 25/litre.

Similar results can be obtained for all other age and sex groups (described in Methods) as well. The visualisation (Fig. 1(a)) of the optimised diet with milk subsidy showed that the requirement of all nutrients could be met, and the diet provided more than two times the EAR for folate, Mg and vitamins A and C. The daily protein requirement could be met by the optimised diet, with milk and milk products constituting 38 % of the total weight of foods in the optimised diet (Fig. 1(b)). The household affordability check suggests that all four optimised diets could be afforded by a family of four members with one earning member with an average wage per day of INR 272 (USD 3·3)^(23)^in Bihar. To afford *Diet4*, 57 % of income must be spent just on food with the specified average wage.

The optimised vegetarian diet for a family of 4 and 2 members with specified composition based on DOT is given in Table 4, and Table 5 shows the average nutrients in the optimised diet for the family. Satisfying all the constraints, a cost of INR 204 was required daily for a family of 4 members to meet their daily nutrient requirements, while a cost of INR 130 was required for a family of 2 members (adult male and adult female). Online supplementary material, Supplemental Table 2 shows the quantities of food items for each family member.

**Table 4:** Quantity of foods for an optimised diet^*^ for a family of 4 members and 2 members

Food items	Amount of food (g)^†^	Amount of food (g)^‡^
Wheat flour atta	226	141
Rice	226	141
Ragi	226	141
Green gram lentil	113	71
Bengal gram lentil	113	71
Amaranth leaves (red)	679	473
Fenugreek leaves	120	60
Palak	120	60
Cow milk	1622	1082
Oil	79	46
Sugar	154	94
Salt	21	16
Potato	200	85
Colocasia	40	20
Onion	40	20
French beans	40	20
Pumpkin (orange)	41	20
Carrot	40	20
Orange	1^*^87	133
Banana	187	133
Papaya	187	133

* Demonstration example generated using DOT tool (https://www.datatools.sjri.res.in/DOT/).^†^

† Quantity of foods for a family of 4 members.^‡^

‡ Quantity of foods for a family of 2 members.

**Table 5:** Nutrient composition of optimised diet^*^ for a family of 4 members and 2 members

Nutrient	Nutrient composition^†^	Nutrient composition^‡^
Ca (mg)	1292	1683
Dietary fibre (g)	36	46
Energy (kcal)	1580	1974
Fat (g)	44	55
Folate (µg)	420	531
Iodine (µg)	156	240
Fe (mg)	25	33
Mg (mg)	663	860
Vitamin B_3_ (mg)	9	12
Protein (g)	52	66
Vitamin B_6_ (mg)	1	2
Vitamin B_2_ (mg)	1	2
Vitamin B_1_ (mg)	1	1
Vitamin A (µg)	2605	3412
Vitamin B_12_ (µg)	1	2
Vitamin C (mg)	42	60
Zn (mg)	10	12

* Demonstration example generated using DOT tool (https://www.datatools.sjri.res.in/DOT/).^†^

† Nutrient composition for a family of 4 members^‡^

‡ Nutrient composition for a family of 2 members

## Discussion

This paper details the development and potential use of diet optimisation tools designed to meet the nutrient requirements of Indians at a minimum cost. The optimisation focused on macronutrients and five essential micronutrients (Fe, vitamin A, iodine, folate, Zn), along with B vitamins, vitamin C, Ca and Mg that can be easily sourced from Indian diets. These nutrients have varying concentrations in the foods that make up the dietary intakes of the Indian population, but data are sparse on the calculated risk of dietary inadequacy of most of these nutrients, since reliable intake data of these nutrients based on diet recalls are currently not available for the country. The tools offer flexibility in the choice of foods and the cost of their purchase by the user’s input. Using the FOP tool, we demonstrate, for example, that the cost of optimised diet for children aged 1–3 years is INR 26·8/day/child (USD 0·32) and that subsidies (50 % subsidy for milk, for example) can reduce this cost by INR 3/day/child. This is a substantial reduction in the cost of an optimised diet by subsidising one food (milk) that has been widely accepted as an important source of multiple nutrients, especially for vulnerable groups such as children. We argue that the tool provides an opportunity for policymakers to try out different permutations of optimised diets, including the effect of hypothetical subsidies that can be offered for different foods. The tool has the potential to support informed, data-driven policy decision-making without requiring knowledge on nutrition and mathematical modelling. There are several optimisation algorithms and tools that have been reported earlier^(27–31)^, but none were specifically developed to consider regional requirements such as the Indian nutrient recommendations^(15)^ and cultural food choices. For instance, the cost of the diet^(27)^ and Optifood^(29)^ tools optimise nutrient intakes using linear programming, but the requirements are not those of the Indian population. The Optifood tool’s need for trained nutritionists limits its usability for implementing bureaucrats who are typically responsible for procuring food provisions for food programmes. A unique feature of the present FOP tool is that it generates a list of locally consumed foods, based on data from the National Sample Survey of India, to optimise food combinations, which facilitates granular site-specific optimisation.

Over the past decade, the idea of micronutrient deficiency being a significant aspect of the ‘triple burden of malnutrition’ in low- and middle-income countries^(32)^ has gained significant attention. To address this nutritional deficiency from a public health perspective, food fortification with micronutrients has become an appealing option for policymakers, industry stakeholders and implementation organisations. However, while food fortification has a role in addressing specific nutrient deficiencies, it is a one-size-fits-all solution, and when excessively used, it can lead to excessive intake of some nutrients, turning what was meant to be a remedy into a potential problem^(33)^. Moreover, the misuse of RDA, which corresponds to the 95th percentile of the nutrient requirement distribution instead of EAR in identifying population-level inadequacy of nutrient intake, amplifies the risk of excessive nutrient intake. The optimisation considered unprocessed, unfortified foods and their combinations. It is observed that various interventions focusing on nutrition have the potential to significantly enhance child welfare in a cost-efficient manner^(34)^. However, this is difficult to optimise without the right tools and is generally considered unaffordable.

According to the 2019 EAT Lancet commission^(35)^, the average recommended diet for rural India would cost INR 333·13 (USD 4)/person/day and reach a cost of INR 1332·54 (USD 16) for a household of 4 members/day. The diet suggested by DOT achieves a diet that is diverse and meets essential macro- and micronutrient requirements with a price of only INR 204 (USD 2·45) for the entire household of 4 members/day and a cost of only INR 130 (USD 1·56) for a household of 2 members/day. This is 15 % of the EAT Lancet commission estimate and indicates the feasibility of achieving affordable and nutritious dietary food combinations without the use of fortification on condition that such foods were available and affordable year-round. For 1–3-year-old children (in the worked example above), the diet suggested by the FOP tool shows that a diverse and adequate diet that meets the essential macro- and micronutrient requirements can be affordably secured at a cost of INR 24 and INR 27/child/day, with and without subsidised milk prices, respectively.

While these costs seem low, they can be substantial per the wages reported from the RBI^(23)^. That is, 72 % of total household income would have to be spent on food for a household of 4 members (2 children, 1 female adult, 1 male adult) with 1 earning member. Even with 2 earning members, more than 35 % of the combined earnings would have to be spent on the recommended diet. Furthermore, for a family of 6 members (2 children, 2 female adults, 2 male adults), the recommended diet is *not* affordable for a household with a single earning member. It is therefore essential to examine income transfers and employment growth to increase purchasing power, alongside possible consumer subsidies for nutrient-dense foods such that appropriate diets become accessible to all. For example, policymakers might explore^(10)^ expanding price subsidies to include other locally produced, nutritionally rich foods under the Targeted Public Distribution System. This could involve decentralising the procurement of various food grains like millets, eggs, soybeans and sorghum based on local needs and providing them at subsidised prices through fair price shops.

Even with optimised use of local ingredients, additional non-diet approaches may be necessary to ensure sufficient intake of Fe and Zn and sometimes Ca, folate, thiamine, riboflavin and niacin^(36)^. The potential for fortification, biofortification and supplementation in such cases should be explored as complements to dietary solutions, not alternatives.

The limitation of the DOT is that the user is compelled to choose from a limited list of foods to fulfil the nutrient requirements. The limitation of the FOP tool is that the National Sample Survey data from which the list of foods to optimise combinations is taken (based on the district and state of choice) are over a decade old and were gathered in 2011–2012. Food choices may have changed over the intervening period. Finally, neither of the tools has the option to add new ingredients to the database.

In conclusion, it is possible to optimise daily dietary food combinations and meet the nutrient requirements of the Indian population while using diverse diets, using the FOP tool and the DOT, to optimise for 17 macro- and micronutrients. The FOP tool can be used by policymakers to identify the foods that need to be available locally by informed decisions, either at subsidised or regular rates, to meet the nutrient requirements of the population. These tools are available in an interactive format online and offer a simple interface for lay users to perform complex linear programming food optimisation algorithms with several different constraints.

## Supporting information

Ayoob et al. supplementary material Ayoob et al. supplementary material

## Supplementary material

For supplementary material accompanying this paper, visit https://doi.org/10.1017/S1368980025100748.
