# Supplementary material for: Optimisation tools for meeting nutrient requirements of Indian children and adults at optimal cost
Source: Public Health Nutr. 2025 Jul 16;28(1):e127. doi: 10.1017/S1368980025100748 (PMC12465075; doi:10.1017/S1368980025100748)
Supplement: Ayoob et al. supplementary material [file S1368980025100748sup001.docx]

**Supplementary Table 1. Estimated Average Nutrient Requirement***

| Nutrients | EAR-child(1-3)yrs | EAR-child(4-6)yrs | EAR-child(7-9)yrs | EAR-women-sedentary | EAR-women-moderate | EAR-women-heavy | EAR-men-sedentary | EAR-men-moderate | EAR-men-heavy | EAR-boys-10-12 | EAR-girls-10-12 | EAR-boys-13-15 | EAR-girls-13-15 | EAR-boys-16-18 | EAR-girls-16-18 |
| --- | --- | --- | --- | --- | --- | --- | --- | --- | --- | --- | --- | --- | --- | --- | --- |
| Energy (kcal) | 1010 | 1360 | 1700 | 1660 | 2130 | 2720 | 2100 | 2710 | 3470 | 2220 | 2060 | 2860 | 2400 | 3320 | 2500 |
| Protein (g) | 9.2 | 12.8 | 19 | 36.3 | 36.3 | 36 | 42.9 | 43 | 43 | 26.2 | 26.6 | 36.4 | 34.7 | 45.1 | 37.3 |
| Fat (g) | 25 | 25 | 30 | 20 | 23 | 30 | 23 | 23 | 39 | 35 | 45 | 50 | 35 | 40 | 35 |
| Fibre (g) | 15.15 | 20.4 | 25.5 | 24.9 | 31.95 | 40.8 | 31.5 | 40.65 | 52.05 | 33.3 | 30.9 | 42.9 | 36 | 49.8 | 37.5 |
| Ca (mg) | 400 | 450 | 500 | 800 | 800 | 800 | 800 | 800 | 800 | 650 | 650 | 800 | 800 | 850 | 850 |
| Zinc (mg) | 2.5 | 3.7 | 4.9 | 11 | 11 | 11 | 14 | 14 | 14 | 7 | 7.1 | 11.9 | 10.7 | 14.7 | 11.8 |
| Iron (mg) | 6 | 8 | 10 | 15 | 15 | 15 | 11 | 11 | 11 | 12 | 16 | 15 | 17 | 18 | 18 |
| Magnesium (mg) | 111 | 131 | 178 | 270 | 270 | 270 | 320 | 320 | 320 | 223 | 214 | 294 | 270 | 338 | 279 |
| Iodine (µg) | 65 | 80 | 80 | 95 | 95 | 95 | 95 | 95 | 95 | 100 | 100 | 100 | 100 | 100 | 100 |
| Vitamin A (µg) | 180 | 240 | 290 | 390 | 390 | 390 | 460 | 460 | 460 | 360 | 370 | 430 | 420 | 480 | 400 |
| Folate (µg) | 90 | 111 | 142 | 180 | 180 | 180 | 250 | 250 | 250 | 180 | 186 | 238 | 204 | 286 | 223 |
| Vit B12 (µg) | 1 | 1 | 2 | 2 | 2 | 2 | 2 | 2 | 2 | 2 | 2 | 2 | 2 | 2 | 2 |
| Vit B1 (mg) | 0.6 | 0.8 | 1 | 1.1 | 1.4 | 1.8 | 1.2 | 1.5 | 1.9 | 1.3 | 1.2 | 1.6 | 1.3 | 1.9 | 1.4 |
| Vit B2 (mg) | 0.8 | 1.1 | 1.3 | 1.6 | 2 | 2.6 | 1.6 | 2.1 | 2.7 | 1.7 | 1.6 | 2.2 | 1.9 | 2.5 | 1.9 |
| Vit B3 (mg) | 6 | 8 | 10 | 9 | 12 | 15 | 12 | 15 | 19 | 12 | 12 | 16 | 13 | 19 | 14 |
| Vit B6 (mg) | 0.8 | 1 | 1.3 | 1.6 | 1.6 | 2.1 | 1.6 | 2.1 | 2.6 | 1.7 | 1.6 | 2.2 | 1.8 | 2.5 | 1.9 |
| Vit C (mg) | 22 | 27 | 36 | 55 | 55 | 55 | 65 | 65 | 65 | 45 | 44 | 60 | 55 | 69 | 57 |

*ICMR-NIN Expert Group on Nutrient Requirement for Indians, Recommended Dietary Allowances (RDA) and Estimated Average Requirements (EAR) – 2020

**Supplementary Table 2.** Quantities of food items for each household member*

| **Food Items** | **Child-Male-1-3 Years (Daily Intake)**  (Amount of Food (g)) | **Child-Female-4-6 Years (Daily Intake)**  (Amount of Food (g)) | **Adult-Male->18 Years (Daily Intake)**  (Amount of Food (g)) | **Adult-Female-NPNL (15-45 Years) (Daily Intake)**  (Amount of Food (g)) |
| --- | --- | --- | --- | --- |
| Wheat flour atta | 35.0 | 51 | 79 | 62 |
| Rice | 35 | 51 | 79 | 62 |
| Ragi | 35 | 51 | 79 | 62 |
| Green gram dhal | 18 | 26 | 40 | 31 |
| Bengal gram dhal | 18 | 26 | 40 | 31 |
| Amaranth leaves (red) | 64 | 143 | 284 | 190 |
| Fenugreek leaves | 30 | 30 | 30 | 30 |
| Palak | 30 | 30 | 30 | 30 |
| Cow milk | 271 | 271 | 541 | 541 |
| Oil | 13 | 21 | 28 | 19 |
| Sugar | 26 | 34 | 53 | 42 |
| Salt | 3 | 3 | 8 | 8 |
| Potato | 57 | 60 | 49 | 36 |
| Colocasia | 10 | 10 | 10 | 10 |
| Onion | 10 | 10 | 10 | 10 |
| French beans | 10 | 10 | 10 | 10 |
| Pumpkin (Orange) | 11 | 10 | 10 | 10 |
| Carrot | 10 | 10 | 10 | 10 |
| Orange | 25 | 30 | 72 | 61 |
| Banana | 25 | 30 | 72 | 61 |
| Papaya | 25 | 30 | 72 | 61 |

*Demonstration example generated using DOT tool (<https://www.datatools.sjri.res.in/DOT/>)
